# Digital maturity: towards a strategic approach

**DOI:** 10.3399/bjgp25X741357

**Published:** 2025-05-02

**Authors:** Trisha Greenhalgh, Rebecca Payne

**Affiliations:** Nuffield Department of Primary Care Health Sciences, University of Oxford, Oxford.; Nuffield Department of Primary Care Health Sciences, University of Oxford, Oxford; Honorary Clinical Senior Lecturer, North Wales Medical School, Bangor University, Bangor.

Ask yourself three questions. First, how would you rate your practice’s digital maturity? Second, where would you like your practice to be on a digital maturity scale? Third, how useful do you find the concept of ‘digital maturity’?

Let’s take the last of these questions first. Sociologists call the period we are currently living in ‘late modernity’. They depict it as characterised by rapid technological change along with a prevailing assumption that the more digitally advanced society becomes, the better.^1^ An aspect of this mindset is the idea that organisations should progress, through recognisable ‘stages’, from the technological Stone Age to a state where technology use is maximised, delivering efficiencies, return on investment, and (in health care) benefits for patients.^2^^–^^6^

If you think this ‘maturational’ model fits poorly with efforts to harness the potential of digital technologies in your own practice, you may be heartened by critiques of it.^7^^–^^9^ Even in commercial businesses, and even more so in institutional organisations like health care, technologisation can lead to ‘organised immaturity’.^9^ This is defined as occurring when an organisation regresses on core Enlightenment ideals that defended individuals’ ability to reflect on their situation, make choices and moral judgements, and challenge norms and institutions in defence of the common good. To the extent that the introduction of particular technologies has made you less able to deliver high-quality clinical care to your patients, you are right to protest that this does not represent progress in any meaningful sense.

In a recent 28-month ethnographic study of the fortunes of 12 UK general practices at varying levels of digital advancement (Remote by Default 2), we found examples, associated with various digital innovations, of unanticipated reductions in work efficiency,^10^ erosion of relational continuity,^11^ concerns about risks to patient safety,^12^ high levels of ‘technostress’ in staff,^13^ unmet training needs,^14^ and the compounding of inequities among vulnerable patients.^15^^,^^16^ Digitalisation of access routes was associated with an inability to access care in some patients with complex needs or multiple markers of disadvantage. These included poverty, homelessness, drug dependence, language barriers, cognitive and literacy challenges, and multimorbidity.^15^^,^^16^ Other researchers have also highlighted widening of inequities as digital access routes become the default.^17^

However, we also documented examples of digital technologies and pathways that improved the efficiency of work processes, sometimes dramatically — for example, when a high-volume back-office function such as flu jab recall was successfully automated or when a new telephony system provided increased capacity and a queueing function.^10^^,^^18^ Furthermore, we found that some (though not all) technologies that were initially experienced as ‘clunky’, clinically somewhat risky, operationally inefficient, or stressful for staff became more widely accepted, useful, and clinically reliable over time. This could occur, for example, as organisational members learnt how to maximise their potential and adapt use cases and work processes to align with what the technology could and could not do, and when product developers modified the technology following user feedback.^18^^,^^19^

The need for such embedding has been demonstrated in multiple research traditions, including Cherns’ early work on sociotechnical workplace design;^20^ Barley’s ethnographic study showing that the same technology introduced in different healthcare settings can meet very different fortunes depending on how roles and workflows are shaped in response;^21^ the more recently emerging traditions of human factors and ergonomics^22^ and human-centred design^23^ in health care; and Campbell *et al*’s call for ‘digital facilitation’ in general practice.^17^

The varied digital fortunes of the 12 practices in the Remote by Default 2 study prompted us to develop a novel taxonomy of digital maturity that recognised that, for some, especially those serving multiply disadvantaged communities, more digitalisation is not always better. Box 1 shows our taxonomy of five ‘ideal type’ practices (ideal in the sense of being a general descriptor to which any actual general practice will approximate). Each of these types has different strengths and weaknesses, and each requires a different kind of support.

**Box 1.** Typology of general practices in relation to digital innovation (adapted under Creative Commons Licence from Greenhalgh *et al*)^18^

**Type**	**Support needs and strategic contribution**
**DIGITAL TRAILBLAZER** — Digital innovation is a core part of the practice’s ethos and identity. In-house human and technical infrastructure is strongly geared to capturing innovations, bringing them into the practice and making them work. Digital technologies are quickly piloted and (if successful) smoothly routinised. Emphasis is typically on efficiency (for example, prompt waiting times). Staff may include digital entrepreneurs with industry links who help to procure and test technologies across a network. The practice population is typically also digitally enabled and skilled (for example, young professionals).	Trailblazer practices could serve as ‘sentinel’ or ‘beacon’ sites to inform policymakers and horizon scanners of novel digital technologies and illustrate how to optimise their use in innovative processes and pathways. Their enthusiasm for digital solutions means they may need reminding and incentivising to ensure that the needs of non-digitally enabled patients are identified and fully addressed.
**DIGITALLY STRATEGIC** — Typically large, well-resourced, and with strong leadership and high organisational innovativeness. Digital technologies are readily identified, introduced, and evaluated as part of a wider strategic vision that also includes, for example, serving particular patient needs, teaching/training, or research. Enthusiasm for particular digital innovations will vary depending on alignment with practice values and patient/staff needs. In some practices (for example, where key subgroups are at risk of being disadvantaged), strategic decisions will tend to favour a relatively technology-light set-up. In others, the needs of digitally less confident patients may be addressed via human intermediaries like digital navigators.	Digitally strategic practices should be supported to identify, obtain, trial, and routinise the technologies they need to achieve their strategic vision, while also addressing other strategic priorities. A key role for policymakers and commissioners is removing barriers to procurement so practices can source the ‘right’ technological solutions (and move on from the ‘wrong’ ones) promptly. These practices’ decisions to withdraw technologies that failed piloting should be respected. Funding may be needed for intermediary roles.
**DIGITALLY REACTIVE** — Digital technologies tend to be introduced in a somewhat piecemeal way — for example, as a ‘fix’ for an immediate problem (overwhelming patient demand), to respond to a policy must-do, or because someone is curious to experiment. Because adoption decisions are mostly non-strategic, there is little sense that new technologies and pathways serve a clear practice mission. There may be a prevailing ethos of ‘firefighting’ and limited acknowledgement of, or investment in, the work of embedding and co-adapting the technology and the work pathway. Patients and staff may be dissatisfied with the overall service.	It is important to identify and address the underlying reason(s) why the practice is not taking a strategic approach, for example, excessive workload, suboptimal staffing and skill mix, weak senior-level commitment, inadequate resources, low agreement on strategic direction, or experiencing policy incentives and must-dos as pointless or perverse. Solutions follow — for example, leadership support, business planning, attention to team relationships, and funding for key staff roles.
**DIGITALLY HESITANT** — The hesitant practice lacks one or more key preconditions for organisational innovation (for example, less well-resourced, lacking strong leadership, weak technological infrastructure, limited in-house technological knowledge, and weak processes for introducing and evaluating innovations). The few digital services that are in place may have been externally selected and imposed (for example, by a network) and may be experienced as clunky and stressful by staff who are neither confident nor adequately trained to get the most out of them.	Digitally hesitant practices need significant support not just to introduce particular technologies but to meet the preconditions for innovation. For example, resources may be needed to optimise the existing technological set-up, train clinical and support staff, and provide protected time for strategic planning. Networking events with (or visits to) digitally strategic practices may help build knowledge and confidence.
**STRATEGICALLY TRADITIONAL** — Typically, a small practice serving a less digitally equipped and digitally capable demographic (sometimes a deprived community with complex health and social care needs). Key patient groups may have a strong preference for (and/or have needs that require) predominantly in-person services. The practice may make selected use of digital technologies (for example, for back-office functions or to allow some patients to order prescriptions online) but are careful to prioritise in-person services for those who need them.	Policymakers should acknowledge that in some contexts digitalisation may worsen inequities and put vulnerable groups at risk. They should support practices serving such populations to provide a traditional, ‘in-person-by-default’ service, including optimising low-tech components such as telephony systems and text messaging, and maximising the potential of digital solutions for back-office (non-patient facing) functions.

The most digitally advanced (‘digital trailblazer’) practices are outliers. They have an unusually high absorptive capacity^24^ for identifying, incorporating, testing, and embedding digital technologies (for example, adequate bandwidth and server capacity with room for expansion, a high level of in-house know-how, extensive past experience introducing similar technologies, robust data systems, and an entrepreneurial culture in which staff are expected to have bold ideas and try them out). Some staff are exceptionally digitally skilled, and the practice may serve an unusually digitally equipped and capable patient population. These practices are likely to be at the forefront of new developments, such as digital scribing and AI-enabled generation of referral letters and safety-netting information.

Much more common is the practice that is ‘digitally reactive’ (the middle category in our taxonomy), doing its best to keep abreast of digital innovations, responding to policy must-dos and cautiously experimenting with new products (typically introduced by individual ‘lone innovators’ among the staff).The digitally reactive practice falls short of a ‘digitally strategic’ approach in which innovations are introduced, evaluated, and, where appropriate, withdrawn as part of a planned, practice-wide strategy. It was heartening that, in the 28 months of our fieldwork, we observed several practices progress from ‘digitally reactive’ to ‘digitally strategic’.

There were several reasons why many digitally reactive practices in our sample were able to shift towards a more strategic approach. They often had moderate absorptive capacity for adopting technological innovations (in other words, an in-house digital infrastructure that was adequate if not advanced; some degree of in-house know-how and a culture of experimentation). This meant that they already possessed, at least to some extent, the organisational preconditions to learn about, introduce, pilot, and evaluate new digital technologies. Through accumulated experiences, they became steadily more confident and capable of addressing digital innovation proactively and systematically.

Additionally, the literature on adoption of innovations in health care identifies networking with other, similar organisations as a powerful impetus to innovation and change.^25^ The Remote by Default 2 study included a (relatively modest) collaborative learning component in which participating practices were linked with one another in workshops and seminars, providing opportunities for mutual support, exchange of resources, and vicarious learning. This input may have added to similar support provided regionally or locally by pre-existing networks (for example, Local Medical Committees, Royal College of General Practitioners faculties, social media, such as GP Facebook groups, and personal relationships). Finally, in some but not all examples, there was local provision for staff training and practice development in relation to digital innovation — for example, from Integrated Care Boards. Such provision varied greatly between localities.

The two least digitally advanced types of practice may, on superficial assessment, be hard to distinguish from one another. Both provide only basic digital services (and, in rare cases, none at all). But the ‘digitally hesitant’ practice, characterised by critically low absorptive capacity (very basic and often fragile digital infrastructure, poor broadband connectivity, and limited in-house knowledge and know-how) as well as weak leadership and absence of strategy, is very different from the ‘strategically traditional’ practice. The latter is typically a ‘Deep End’ inner city practice or sited in a traditional remote and rural community, serving a population for whom digital services are unsuitable or low priority. Whereas the digitally hesitant practice may benefit from intensive organisational support and technological capacity building to meet the preconditions for innovation, the strategically traditional practice needs acknowledgement that its low-tech approach is appropriate in the circumstances, and a tailoring of incentives and support to ensure that it is not penalised for its Enlightenment-informed strategy.

To distinguish between the two very different ‘low-tech’ practices (strategically traditional versus digitally hesitant), three questions may be useful. First, what is the evidence that the dearth of digital technologies and processes in the practice is the result of strategic decisions rather than to limitations of leadership, planning, resources, and so on? Second, to what extent does the focus on in-person interactions serve the practice population (and vulnerable subgroups within it) rather than disadvantaging them? Third, is the practice maximising appropriate areas of digitalisation (for example, for back-office functions), while ensuring that patient-facing interactions remain relatively technology-light?

Our findings suggest that the current policy of digital innovation in the NHS needs some nuance.^26^ Different levels of digital maturity need different approaches (Box 1, column 2). Of particular note is the finding that while digitally reactive practices can often, over a relatively short period of time, transition to digitally strategic without bespoke support, the digitally hesitant practice typically needs substantial input to meet the preconditions for digital innovation. Also, there is no formula that will tell policymakers how to balance the efficiency-focused drive for digital transformation of general practice generally with an equity-focused recognition and support for traditional (low-tech) practices where appropriate. We hope that taxonomy presented in Box 1 will inform reflections and dialogues around how to strike this balance in practice.

It is easy to feel powerless in the face of technological over-enthusiasm from politicians and lofty promises from suppliers. As practices and practitioners, informed by the communities we serve, we must retain the power to shape our own technological destinies. For some practices, that will involve blazing a trail into a technologically augmented future. But for others, making a strategic choice to stick to traditional modes of care provision will be equally appropriate and must be equally supported.
